# LRIT3 is Required for Nyctalopin Expression and Normal ON and OFF Pathway Signaling in the Retina

**DOI:** 10.1523/ENEURO.0002-20.2020

**Published:** 2020-02-06

**Authors:** Nazarul Hasan, Gobinda Pangeni, Thomas A. Ray, Kathryn M. Fransen, Jennifer Noel, Bart G. Borghuis, Maureen A. McCall, Ronald G. Gregg

**Affiliations:** 1 Department of Biochemistry and Molecular Genetics, University of Louisville, Louisville, KY 40292; 2 Department of Ophthalmology and Visual Sciences, University of Louisville, Louisville, KY 40292; 3 Department of Anatomical Sciences and Neurobiology, University of Louisville, Louisville, KY 40292

**Keywords:** CSNB, glutamate dynamics, LRIT3, night blindness, nyctalopin

## Abstract

The first retinal synapse, photoreceptor→bipolar cell (BC), is both anatomically and functionally complex. Within the same synaptic region, a change in presynaptic glutamate release is sensed by both ON BCs (DBCs) via the metabotropic glutamate receptor 6 (mGluR6), and OFF BCs (HBCs) via ionotropic glutamate receptors to establish parallel signaling pathways that preferentially encode light increments (ON) or decrements (OFF), respectively. The synaptic structural organization of ON and OFF-type BCs at the photoreceptor terminal differs. DBCs make an invaginating synapse that contains a diverse but incompletely understood complex of interacting proteins (signalplex). HBCs make primarily flat contacts that contain an apparent different set of proteins that is equally uncharacterized. LRIT3 is a synaptic protein known to be essential for ON pathway visual function. In both male and female mice, we demonstrate that LRIT3 interacts with and is required for expression of nyctalopin, and thus TRPM1 at all DBC dendritic tips, but DBC signalplex components are not required for LRIT3 expression. Using whole-cell and multielectrode array (MEA) electrophysiology and glutamate imaging, we demonstrate that the loss of LRIT3 impacts both ON and OFF signaling pathway function. Without LRIT3, excitatory input to type 1 BCs is reduced, as are the visually evoked responses of many OFF retinal ganglion cells (RGCs). We conclude that the absence of LRIT3 expression disrupts excitatory input to OFF BCs and, thus disrupts the normal function of OFF RGCs.

## Significance Statement

At the first visual synapse, photoreceptor cells signal to two distinct bipolar cell (BC) populations, one characterized by a depolarizing response to light onset (ON or DBCs), the other by a hyperpolarizing response (OFF or HBCs). The DBC light response depends on a G protein-coupled metabotropic glutamate receptor (mGluR) and associated protein complex, known as the DBC signalplex. Loss of function mutations in signalplex proteins lead to loss of scotopic visual function, and causes congenital stationary night blindness (CSNB) in humans. Here we show how the loss of LRIT3, a previously identified signalplex protein, not only prevents functional assembly of the DBC signalplex, but also alters visual function through both ON and OFF signaling pathways.

## Introduction

Light signaling starts in the retina when photoreceptors detect a luminance increase, hyperpolarize, and decrease tonic glutamate release. Differences in rod and cone photoreceptor light sensitivity creates parallel channels that encode visual signals under dim and bright illumination, and signal through rod bipolar cells (BCs) and multiple types of cone BCs, respectively. The parallel cone BC pathways comprise depolarizing (ON or DBCs) and hyperpolarizing (OFF or HBCs) BCs that selectively encode light increments and decrements, respectively. This difference in response polarity results from the type of glutamate receptors expressed. Rod and cone ON BCs signal via metabotropic glutamate receptor 6 (mGluR6; Kaneko and Saito, 1983; Saito and Kaneko, 1983; Slaughter and Miller, 1983; Borghuis et al., 2014), whereas OFF BCs signal via AMPA/kainate receptor (Kaneko and Saito, 1983; Saito and Kaneko, 1983; Slaughter and Miller, 1983; DeVries and Schwartz, 1999; Borghuis et al., 2014; Ichinose and Hellmer, 2016).

Mutations affecting rod photoreceptor presynaptic proteins generally disrupt the invaginating synapse and ribbon structure. These presynaptic disruptions correlate with ectopic extensions of DBC and HC dendrites into the ONL (Ball et al., 2002; Haeseleer et al., 2004; Mansergh et al., 2005; Chang et al., 2006; Wycisk et al., 2006). In contrast, most mutations affecting postsynaptic DBC signalplex proteins do not alter the morphology of the invaginating ribbon synapse, although they disrupt light signaling due to their role in signal transduction (Masu et al., 1995; Ball et al., 2003; Morgans et al., 2009; Koike et al., 2010; Peachey et al., 2012; Neuillé et al., 2017). Many elements of the DBC signalplex are required for normal glutamate signaling, including: mGluR6, TRPM1, GPR179, nyctalopin, RGS7, RGS11, R9AP, Gα0, Gβ13, Gβ5, and LRIT3.

Most studies agree that functional interactions within the mGluR6 signalplex are similar in rod and cone DBCs, and that there is a dependence-hierarchy of DBC signalplex protein expression. For example, TRPM1 expression depends on expression of nyctalopin (Pearring et al., 2011), and GPR179 is required for expression of RGS7, RGS11, and R9AP (Sarria et al., 2016). Absence of the expression of RGS7, RG11 and R9AP does not disrupt expression of mGluR6, TRPM1, or nyctalopin (Ray et al., 2014; Sarria et al., 2016). Leucine-rich repeat (LRR) containing protein LRIT3 is a recently discovered member of the signalplex with some structural and functional similarities to nyctalopin, including an apparent extracellular LRR domain. LRIT3 also contains two additional domains, a Fn3 and IgG, which are known to interact with many proteins (Kim et al., 2012). Functionally, loss of the protein leads to a no b-wave phenotype. However, unlike other known members of the RBC signalplex, LRIT3 is expressed in rod photoreceptors, and when its expression is restored in *Lrit3^-/-^* mouse rods, scotopic retinal function is rescued (Hasan et al., 2019). These observations and the structure of LRIT3 raise the question of whether LRIT3 interacts with nyctalopin. Further, LRIT3’s expression in photoreceptors suggests it could influence pre-, as well as postsynaptic signaling complexes (Hasan et al., 2019). To examine the impact of the loss of LRIT3 on retina function, we created an *Lrit3^-/-^* mouse line and examined expression of key signalplex proteins and downstream retinal function.

Our data demonstrate that LRIT3 is required for nyctalopin localization to the DBC dendritic tips. LRIT3 is required for mGluR6 and GPR179 localization in in cone DBCs but not rod BCs. In addition to the expected lack of visual function in DBCs and ON retinal ganglion cells (RGCs), we found that visual responses of *Lrit3^-/-^* HBCs and OFF RGCs were significantly reduced.

Our results demonstrate that LRIT3 is the first protein whose absence impacts both ON and OFF signaling pathways without gross defects in the photoreceptor synaptic architecture. Because LRIT3 is necessary for assembling the postsynaptic DBC signalplex and for normal visual function signaling in both DBCs and HBCs, loss of LRIT3 impacts visual responses of nearly all RGCs.

## Materials and Methods

### Animals

All procedures were performed in accordance with the Society for Neuroscience policies on the use of animals in research and the University of Louisville Institutional Animal Care and Use Committee. Animals were housed in the University of Louisville AAALAC approved facility under a 12/12 h light/dark cycle. The mouse line described in these studies, *Lrit3^emrgg1^*, is referred to as *Lrit3^-/-^* throughout. The phenotypes of all the other lines have been published. *Trpm1^-/-^* (*Trpm1^tm1Lex^*, Shen et al., 2009), *Grm6^-/-^* (Masu et al., 1995), *Nyx^nob^* (Gregg et al., 2003), *GPR179^nob5^* (Peachey et al., 2012), *TgEYFP-Nyx* (Gregg et al., 2005), *MitoP-CFP* (Misgeld et al., 2007), and *TgVsx1-cerulean* (Hoon et al., 2015). All lines were backcrossed to C57BL/6J for at least 10 generations. The heterozygous lines of all these mice have no detectable phenotype, e.g., are indistinguishable from C57BL/6J; therefore, we used C57BL/6J, and heterozygous littermates as controls. Animals of either sex were used in all experiments. For all procedures, mice were anesthetized with an intraperitoneal or subcutaneous injection of ketamine/xylazine solution (127/12 mg/kg, respectively) diluted in normal mouse Ringer’s or killed using CO_2_ according to AVMA guidelines.

### Generation of *Lrit3^-/-^*mice with zinc finger nucleases (ZFNs)

C3H/HeNTac/C57BL/6NTac hybrid embryos (363) were injected with 10 ng/μl *Lrit3* ZFN mRNA and 254 viable embryos were implanted into nine Swiss Webster recipient mothers. Tail biopsies from offspring were collected and genomic DNA isolated using Direct Tail PCR solution (Thermo Scientific) supplemented with 0.2 μg/ml proteinase K (Thermo Scientific). Primers (5′- TAACCTGGGCATAGCCTGTC-3′; 5′-AAGGTCCAGGAAGGAGAAGG-3′) were used to amplify the ZFN targeted region (chr3:129503565, mm9). PCR fragments were either sequenced directly or cloned into the TopoBlunt vector (Invitrogen) and at least 10 clones sequenced. The *Lrit3^-/-^* allele was backcrossed onto C57Bl/6J mice for 10 generations.

### Antibodies

Guinea pig anti-LRIT3 and rabbit anti-TRPM1 antibodies were generated by immunizing animals with peptides (LRIT3:GELWTRRHRDDGERLLLC; TRPM1: SVVPEGQNTQQEKRSAETE) conjugated to KLH, by Biosynthesis Inc. Antibody, dilution and source are as following: LRIT3, 1:1000 (Hasan et al., 2019); TRPM1, 1:1000 (Hasan et al., 2019); Pikachurin, 1:2000 (Wako Chemicals; Sato et al., 2008); mGluR6, 1:2000 (gift, Dr. Kirill Martemyanov; Cao et al., 2011); and GFP for EYFP, 1:2000 (Cell Signaling catalog #2555). The specificity of the LRIT3 and TRPM1 antibodies were validated by comparing immunostaining in control and *Lrit3^-/-^* and *Trpm1^-/-^* mice, respectively.

### Cell culture, transfection, co-immunoprecipitation (IP), and immunoblotting

Human embryonic kidney (HEK293T) cells were cultured in high-glucose DMEM supplemented with 10% fetal bovine serum, 2 mM L-glutamine, 50 IU/ml penicillin, and 50 μg/ml streptomycin. Cells were seeded on 60-mm culture dishes 1 d before transfection and transfected with a nyctalopin tagged with EYFP, and LRIT expression plasmids, using jetPrime reagent (Polyplus-transfection) according to the manufacturer’s instructions. 48 h after transfection, cells were harvested in NP-40 lysis buffer (50 mM Tris, 150 mM NaCl, 2 mM EDTA, and 1% Nonidet P40, pH 8.0, supplemented with protease inhibitor cocktail; Sigma-Aldrich) and disrupted by rotating for 45 min at 4°C followed by sonication. Cell debris was removed by centrifugation at 17,000 × *g* for 15 min at 4°C, and the supernatant was collected and protein quantified using the Bradford reagent (Bio-Rad).

Co-IP was performed as described (Hasan et al., 2016). Transfected HEK cell lysates (500 μg total protein in 200 μl) were precleared by incubating with 10 μl Dynabeads protein G (Invitrogen, ThermoFisher Scientific) at 4°C for 1 h. Precleared lysates were incubated with 5–10 μg of anti-LRIT3 antibody overnight at 4°C on an orbital rocker. A total of 45 μl of Dynabeads protein G was added and incubated for 2 h at 4°C. Dynabeads were collected and washed four times with Tris-buffered saline containing 0.3% Tween 20, and protein complexes eluted with 40 μl of 4× LDS loading buffer by incubation at 70°C for 10 min, loaded and analyzed on 4–12% NuPAGE gels (Invitrogen, ThermoFisher Scientific), transferred to PVDF membranes, and blocked with Odyssey Blocking buffer. Membranes were incubated with primary antibodies (guinea pig anti-LRIT3; 1:1000 and rabbit anti-GFP; 1:2000), diluted in Odyssey Blocking buffer, and washed four times with TBS containing 0.1% Tween 20 (TBST). After incubating with IRDye800 CW and IRDye680 CW-conjugated secondary antibodies diluted in Odyssey Blocking buffer (LI-COR), membranes were washed four times with TBST. Protein bands were visualized by scanning the membranes in an Odyssey Infrared Imaging System (LI-COR) using both 700- and 800-nm channels.

### Retina preparation for immunohistochemistry

Mice were killed by CO_2_ inhalation followed by cervical dislocation. Eyes were enucleated and the cornea and lens removed. The retina was dissected in PBS (pH 7.4) and fixed for 15–30 min in PBS containing 4% paraformaldehyde, then washed in PBS for 5 min, and cryoprotected in a graded series of sucrose solutions (5, 10, 15, and 20% in PBS) and finally in OCT:20% sucrose (2:1). The retinas were then frozen by immersion in an isopentane bath immersed in liquid nitrogen. Transverse 18-μm sections were cut on a cryostat (Leica Biosystems) and mounted on Superfrost Plus slides (ThermoFisher Scientific). Slides were stored at –80°C until used in immunohistochemistry experiments.

Immunohistochemistry methods have been described (Hasan et al., 2016). Briefly, slides were dried at 37°C for 30 min, rinsed in PBS for 5 min, and then in PBX (PBS + 0.5% Triton X-100) for 5 min. Sections were incubated in blocking buffer (PBX + 5% normal donkey serum) for 1 h followed by overnight incubation with primary antibody in blocking buffer. Sections were washed 3 × 10 min in PBX, then incubated with secondary antibody diluted in blocking buffer for 1 h. Sections were washed 2 × 10 min in PBX and 1 × 10 min in PBS. Coverslips were mounted to slides using Vectashield (Vector Laboratories). Sections were imaged on an FV-3000 Confocal Microscope (Olympus) and corrected for contrast and brightness using Fluoview Software (Olympus) or Photoshop (Adobe Systems). Images shown in the figures are representative, of multiple images per section (typically two to three per section and four sections per slide, from at least three slides generated from each retina. Retinas from at least three mice are processed and examined. We typically collect oversampled *z*-stacks and the images shown are a representation of a flattened *z*-stack.

### Electroretinography

ERGs methods have been described (Ray et al., 2014). Briefly, mice were dark adapted overnight and anesthetized with a ketamine/xylazine solution (127/12 mg/kg, respectively) diluted in normal mouse Ringers and prepared for ERG recordings under dim red light. Pupils were dilated and accommodation relaxed with topical applications of 0.625% phenylephrine hydrochloride and 0.25% Tropicamide and the corneal surface anesthetized using 1% proparacaine HCl. Body temperature was maintained via an electric heating pad (TC1000 Temperature control, CWE Inc.). A clear acrylic contact lens with a gold electrode (LKC Technologies Inc.) was placed on the cornea and wet with artificial tears (Tears Again, OCuSOFT). Ground and reference needle electrodes were placed in the tail and on the midline of the forehead, respectively. For scotopic responses, flashes (from –3.6 to 2.1 log cd s/m^2^) were presented to dark adapted animals. For photopic responses the animals were light adapted (20 cd/m^2^) for 5 min and test flashes (from –0.8 to 1.9 log cd s/m^2^) were presented on this rod saturating background.

### Rod BC recordings

Methods for the preparation of retinal slices and whole cell recordings from BCs have been described (Ray et al., 2014). Briefly, isolated retinas were placed on nitrocellulose paper (Millipore Sigma) and ∼200-μm transverse slices were prepared using a tissue slicer, and placed in a heated recording chamber, which was constantly superfused with oxygenated Ringer’s at 34–35°C. Recording electrodes with resistance measured between 6 and 9 MΩ were filled with Cs- gluconate intracellular solution (20 mM CsCl, 107 mM CsOH, 107 mM D-gluconic acid, 10 mM NaHEPES, 10 mM BAPTA, 4 mM ATP, and 1 mM GTP); 1% sulforhodamine was included in the intracellular solution to visualize and classify the cell based on its morphology (Ghosh et al., 2004).

Inhibitory blockers [1 μM strychnine, 100 μM picrotoxin, and 50 μM 6-tetrahydropyridin-4-yl methylphosphinic acid (TPMPA)] were included in bath solutions as was L-AP4 (4 μM) to saturate mGluR6 receptors.

OFF cone BC somas were targeted for whole cell patch clamp recording in Vsx1-cerulean reporter mice where type 1 and 2 HBCs (BC1 and BC2) are sparsely labeled (Hoon et al., 2015). Only BC1s with an input resistance ∼1 GΩ and access resistance <25 MΩ were used for recording and were voltage clamped at +50 mV (Nawy, 2004; Shen et al., 2009). A Picospritzer II (Parker Instrumentation) was used to pressure apply drugs onto BC dendritic tips located in the outer plexiform layer (OPL). The drugs used were: the mGluR6 receptor antagonist α-cyclopropyl-4- phosphonophenylglycine (CPPG; 0.6 mM) or kainate (50 μm) to activate kainate receptors on HBCs. All reagents were purchased from Sigma-Aldrich, except for L-AP4 and kainate, which were purchased from Tocris Bioscience. Clampfit 10.2 was used for offline analyses of data. Currents were filtered off-line using a 20-Hz eight-pole Bessel low-pass filter.

### Glutamate imaging

To broadly target iGluSnFR expression to RGCs and amacrine cells, AAV2/1.*hSynapsin*.iGluSnFR in suspension was injected into the mouse eye, intravitreally (0.8–1.0 μl, 0.8–3.0 × 10^13^ vg/μl). Animals were killed 14–21 d after AAV injection and the retinas prepared as described (Borghuis et al., 2013). Isolated retinas were mounted photoreceptor-side down on a nitrocellulose filter paper disk (Millipore Sigma), with 1.0-mm-diameter holes for visual stimulation through the condenser, fixed in a perfusion chamber on a custom-built two-photon fluorescence microscope. Tissue was continuously perfused with oxygenated Ames medium at ∼6 ml/min; 34–36°C. Changes in iGluSnFR fluorescence, which represent the binding of glutamate to iGluSnFR, were measured as described (Borghuis et al., 2013), using a 60×, 1.0 NA, LUMPlanFl/IR objective (Olympus) and an ultrafast pulsed laser (Chameleon Ultra II; Coherent) tuned to 915 nm.

The visual stimulus used in imaging experiments comprised a contrast reversing spot (150 μm in diameter for BCs, 300 μm for GCs; 100% Michelson contrast; 1-Hz temporal modulation; 5-s duration) on a mid/high photopic intensity background (λmax = 395 nm; 5.8 × 10^4^ photons/μm^2^/s). Images (512 × 128 pixels) were acquired at 16 frames/s; line scans were collected at 2 kHz and presented down-sampled to 0.5 kHz. Fluorescence responses were quantified using custom algorithms in MATLAB (MathWorks).

### RGC and off BC whole-cell recordings from retinal whole-mounts

Genetically identified OFF BC1 cells were recorded in the whole-mount retina of MitoP-CFP transgenic mice on *Lrit3^-/-^* or *Lrit3^+/-^* backgrounds. α-Type ON and α- and δ-type OFF ganglion cells were targeted for recording based on soma size in *Lrit3^-/-^* and *Lrit3^+/-^* whole-mount retina; cell type was verified *post hoc* using two-photon fluorescence imaging of sulforhodamine 101 filled cells as well as signature features of their recorded current responses. Visually-evoked excitatory and inhibitory currents of BCs and RGCs were recorded in whole-cell configuration at the reversal potential for chloride (–69 mV) and cations (0 mV), respectively, using conventional methods (Multiclamp 700B, Digidata 1550, PClamp10; MDS Analytical Technologies) and cesium-based internal pipette solution (90 mM cesium methanesulfonate, 5 mM TEA-Cl, 10 mM HEPES, 10 mM BAPTA, 3 mM NaCl, 2 mM QX-314, 4 mM ATP-magnesium salt, 0.4 mM GTP-sodium salt, and 10 mM Tris-phosphocreatine; pH 7.3, ∼284 mOsm). OFF BC membrane voltage responses were recorded in current clamp, using potassium-based internal solution (110 mM potassium methane sulfonate, 10 mM HEPES, 0.1 mM EGTA, 5 mM NaCl, 4 mM ATP-magnesium salt, 0.4 mM GTP-sodium salt, and 10 mM Tris-phosphocreatine; pH 7.3, ∼284 mOsm). Data were analyzed using custom algorithms in MATLAB.

### Multielectrode array (MEA) recordings of RGCs

Procedures for recording extracellular spiking activity of RGCs, using the MEA have been published (Peachey et al., 2017). Mice were dark-adapted overnight, deeply anesthetized with IP injection of ketamine/xylazine and killed by cervical dislocation under dim red light. The retinas were dissected under dim red light in oxygenated Ringer’s solution. The vitreous was removed by incubation (10 min) in Ringer’s solution containing collagenase (12 U/μl) and hyaluronidase (50 U/μl; Worthington Biochemicals). Pieces in dorsal and ventral retina were dissected (2 × 2 mm) and placed ganglion cell side down on a sixty electrode MEA (60MEA200/30Ti; Multi Channel Systems). Retinal pieces were covered with a transparent cell culture membrane (ThermoFisher Scientific) and held in place with a platinum ring. The recording chamber was continuously perfused with oxygenated Ringer’s solution 34–35°C throughout the experiment.

Before recording, the preparation was allowed to settle for ∼1 h in darkness. Spontaneous activity under dark-adapted conditions was recorded for 10 min followed by 10 or 20 trials of full-field light stimulation (5 s/2 s or 20 s/10 s interstimulus/stimulus interval, respectively). Light-adapted responses were recorded after 5 min of adaptation to a background of 3.01 cd/m^2^. Three flash intensities were used under dark- and for light-adapted conditions and presented in order of increasing luminance (scotopic levels: 0.01, 0.03, 1.58 cd/m^2^ and photopic levels: 2.71, 14.7 and 303 cd/m^2^).

Signals were bandpass filtered (80–3000 Hz) and digitized at 25 kHz (MC Rack software; Multi Channel Systems). When electrodes recorded spikes from more than one RGC, we sorted spikes using a principal components analysis (Offline Sorter; Plexon). Sorted units were exported, spikes binned (50 ms), and their spontaneous and visually evoked responses were analyzed (NeuroExplorer; Nex Technologies). Spontaneous activity was examined for the presence of rhythmic activity using a power spectral density FFT analyses (NeuroExplorer) with 4096 frequency values and a Hann window function. The mean FFT was smoothed using a Gaussian filter with a bin width of 30 ms. The frequency of the FFT peak was plotted as a function of power in arbitrary units (A.U.). Using custom scripts, we defined light evoked responses as a response with a peak firing rate that was >+10 SEM above mean spontaneous. The time to peak (TTP) of the evoked response was defined as the time after stimulus onset when the peak firing rate reached it maximus.

Raster plots show responses to each stimulus presentation and peristimulus time histograms (PSTHs) represent the average spiking rate across all stimulus presentations.

### Statistical Analyses

Prism 8.2 software (GraphPad Software, Inc.) was used to perform the statistical analyses as suited for the necessary comparisons: two-way repeated measures ANOVAs, two-way ANOVAs, one-way ANOVAs, or *t* tests. Tukey *post hoc* tests were used when appropriate. Statistical significance was determined at *p* < 0.05. When comparing peak firing rate and TTP data, Kruskal–Wallis tests were used. The specific tests used are indicated in the figure legends.

## Results

The absence of LRIT3 causes complete congenital stationary night blindness (cCSNB) in humans and mice (Ray, 2013; Zeitz et al., 2013; Neuillé et al., 2014). We now report a likely mechanism of action and the downstream consequences of its absence.

### LRIT3 is required for expression of and interacts with nyctalopin

**Figure 1.**
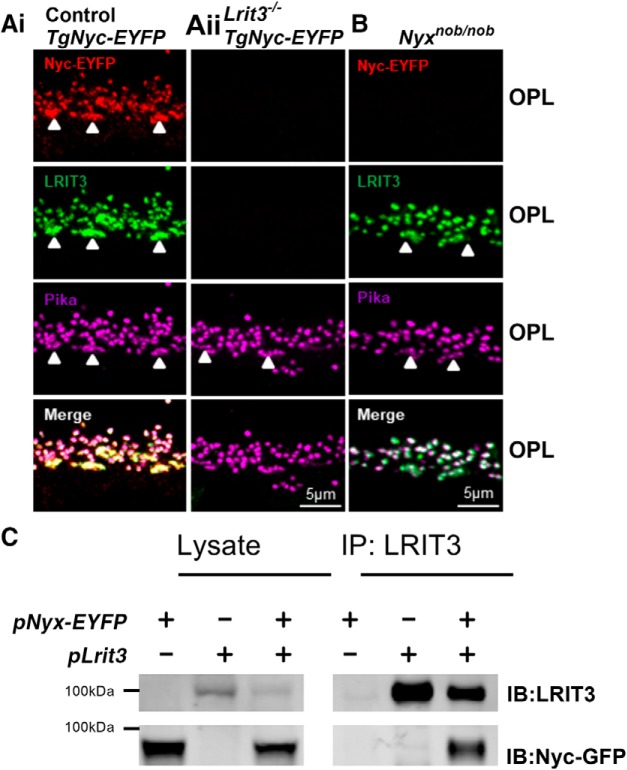
LRIT3 interacts with and is required for nyctalopin expression. ***A***, Confocal fluorescence image of EYFP-nyctalopin (red), LRIT3 (green) and pikachurin (magenta) staining in (***Ai***) control and (***Aii***) *Lrit3^-/-^/TgNyc-EYFP* retinas. Nyctalopin-EYFP is not expressed in the *Lrit3^-/-^/TgNyc-EYFP* retina. Characteristics of the *Lrit3^-/-^* mouse are shown in Extended Data Figures 1-1, 1-2. ***B*,** In *Nyx^nob^* retinas that lack nyctalopin, LRIT3 is expressed and localized to the dendrites of the rod and cone DBCs (marked by arrowheads). Examples show representative images of data from at least three mice. ***C***, LRIT3 and nyctalopin interact in HEK293 cells. HEK293 cells were transfected with expression plasmids *pLrit3*, *pNyx-EYFP* or both, and immunoblotted with antibodies against LRIT3 and EYFP. Lanes for the lysates show that the expected proteins were expressed. The remaining lysate was immunoprecipitated (IP) with antibodies against LRIT3 and the precipitates analyzed by western blotting. An asterisk indicates a non-specific band. Blots are representative of at least three experiments.

We and others have observed that TRPM1 is not expressed in *Lrit3^-/-^* retina (Neuillé et al., 2015; Hasan et al., 2019), and it has been shown that the expression and correct localization of TRPM1 to the photoreceptor to BC synapse depends on the expression of nyctalopin (Pearring et al., 2011). This raises the question; does TRPM1 expression depend on LRIT3 directly or is its expression lost due to a concomitant loss of nyctalopin? To address this, we examined the expression pattern of nyctalopin in *Lrit3^-/-^* and control retinas. Because there are no nyctalopin antibodies we crossed and backcrossed *Lrit3^-/-^* mice to a transgenic mouse line (*TgEYFP-Nyc*) that expresses an EYFP tagged nyctalopin fusion protein (EYFP-nyctalopin) in ON BCs (Gregg et al., 2005). Consistent with the published data, control *TgEYFP-Nyc* retina showed immunopositive expression of EYFP-nyctalopin in the OPL, where it co-localized with both LRIT3 and the synaptic marker, pikachurin (Fig. 1*Ai*), consistent with localization on the dendritic tips of rod and cone DBCs. In *Lrit3^-/-^/TgEYFP-Nyc* retinas EYFP-nyctalopin expression was absent (Fig. 1*Aii*), although these mice carried a copy of the EYFP-nyctalopin transgene. This result shows LRIT3 is required for nyctalopin expression at the DBC dendritic tips, in addition to TRPM on DBCs and mGluR6, GPR179 and RGS complex proteins in cone ON BCs (Extended Data Figs. 1-1, 1-2; Ray, 2013; Neuillé et al., 2015; Hasan et al., 2019).

10.1523/ENEURO.0002-20.2020.f1-1 Extended Data Figure 1-1 LRIT3 is required for normal ERGs and is expressed in the OPL. ***A***, Schematic of the *Lrit3* gene indicating the target region (*) for the ZFN and resulting 40 bp deletion in the *Lrit3^-/-^* mouse line. ***B***, Electroretinograms of control (black symbols) and *Lrit3^-/-^* (red symbols) mice under scotopic and (***C***) photopic conditions. Example response waveforms for five (scotopic) and three (photopic) luminance steps are shown, as well as summary data for all luminance steps. The *Lrit3^-/-^* mice have a normal a-wave that is similar in amplitude control mice (*p* > 0.05 at all flash intensities, *t* tests followed by Bonferroni correction for multiple testing). In contrast, *Lrit3^-/-^* mice lack the b-wave under both scotopic and photopic conditions. The control b-wave amplitude is significantly greater than 0 at all flash intensities (*p* > 0.05 one sample *t* tests followed by Bonferroni correction for multiple testing). ***D***, DIC (left) and immunohistochemical staining for LRIT3 in transverse sections from the control mouse retina. ***E***, LRIT3 staining of OPL of control and *Lrit3^-/-^* retinas. ***F***, Western blotting for LRIT3 and a loading control β-actin in control and *Lrit3^-/-^* retinas. These data validate the specificity of the LRIT3 antibody is specific. ***G***, TRPM1 (green) is mislocalized in *Lrit3^-/-^* OPL and Pikachurin expression is the same as control. These are representative images of data from at least four mice. OS, outer segments; INL, inner nuclear layer; GC, ganglion cell layer. Download Figure 1-1, TIF file.

**Figure 2:**
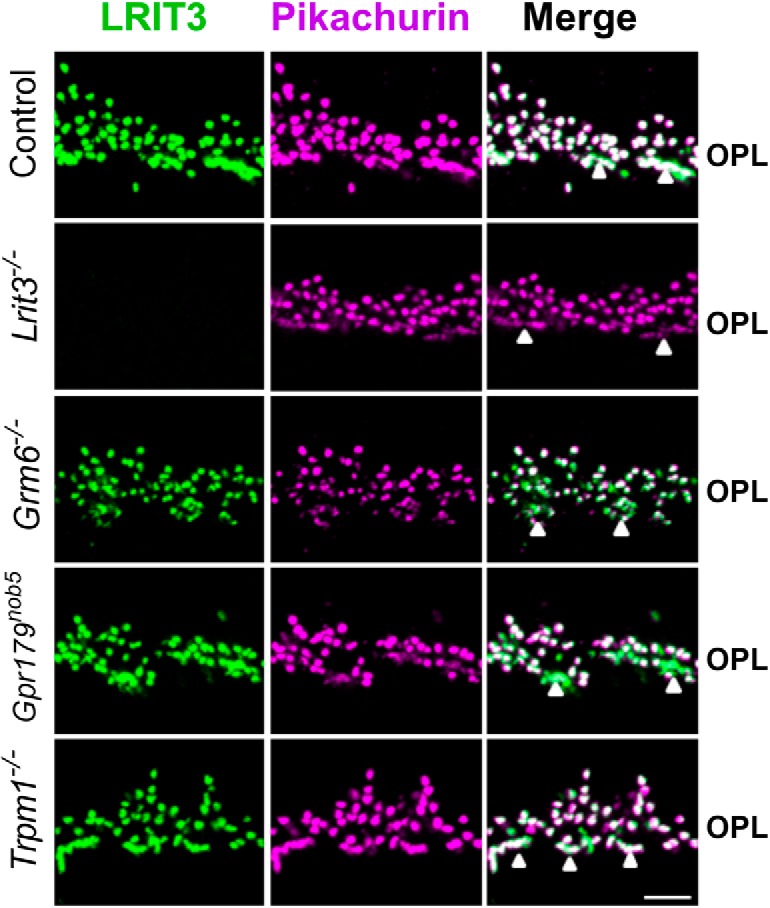
LRIT3 and Pikachurin are expressed normally in the OPL of *Grm6^-/-^*, *Gpr179^nob5^*, and *Trpm1^-/-^*mouse retinas. Confocal fluorescence images of retinas from control, *Lrit3^-/-^*, *Grm6^-/-^*, *Gpr179^nob5^*, and *Trpm1^-/-^* mouse retinas, immunostained for LRIT3 (green) and pikachurin (magenta). The merged images show that LRIT3 and Pikachurin co-localize. Staining for CACNA1F, PNA, and mCAR (cone arrestin) are shown in Extended Data Figure 2-1. Arrowheads indicate cone terminals. Scale bar = 5 μm.

10.1523/ENEURO.0002-20.2020.f1-2 Extended Data Figure 1-2 The absence of LRIT3 has differential effects on rod and cone DBC signalplex proteins. Immunohistochemical staining for GPR179, mGluR6, Gβ5, RGS7, RGS11, and R9AP show punctate staining at the dendritic tips of both rod and cone (large clusters at the base of the OPL and indicated by arrowheads) DBCs in control mice. In *Lrit3^-/-^* mice these proteins are localized on the rod DBC dendritic tips but are absent from the cone DBCs. Note the lack of the large clusters at the bottom of the OPL. Scale bar = 5 μm. INL, inner nuclear layer. Scale bar = 5 μm. Download Figure 1-2, TIF file.

We next tested whether LRIT3 expression depends on nyctalopin by examining its expression in *Nyx^nob^* mutant mice, which lack nyctalopin expression. *Nyx^nob^* and control retina (Fig. 1*B*) have a similar distribution of immunopositive profiles in the OPL for both LRIT3 and pikachurin, and these profiles co-localize with the rod and cone DBC (arrowheads) tips. In contrast, TRPM1 expression is absent on *Lrit3* ^-/-^ DBC dendritic tips (Extended Data Fig 1-1*G*), although expression is present in the soma as reported (Pearring et al., 2011). We conclude that nyctalopin expression depends on LRIT3 expression, and that the loss of TRPM1 from the dendritic tips of *Lrit3* ^-/-^ rod and cone DBCs results from the loss of nyctalopin expression. Given that LRIT3 expression in rods can restore function to *Lrit3^-/-^* rod BCs (Hasan et al., 2019) we determined whether LRIT3 and nyctalopin interact. We expressed each protein alone or in combination in HEK293 cells, prepared cell lysates and did IPs. Cell lysates and immunoprecipitated proteins were run on western blots and probed using antibodies to LRIT3 and EYFP-nyctalopin (Fig. 1*C*). After IP of LRIT3, staining for EYFP-nyctalopin is only present when both proteins are expressed, indicating the two proteins interact in this heterologous expression system.

### LRIT3 expression is independent of other DBC signalplex components

The results from many labs suggest that there is a complex interdependency among the DBC signalplex components mGluR6, GPR179, TRPM1, and nyctalopin (for review, see Gregg et al., 2014). To examine LRIT3 in this interdependency, we determined whether either mGluR6, GPR179 or TRPM1 were required for LRIT3 expression by staining for LRIT3 in *Grm6^-/-^*, *Gpr179^-/-^*, and *Trpm1^-/-^* mouse retinas. Similar to nyctalopin, LRIT3 expression was the same in control and each mutant mouse retina (Fig. 2). Thus, LRIT3 trafficking and localization is independent of mGluR6, GPR179, TRPM1, and nyctalopin expression at the synapse between photoreceptors and BCs (Fig. 2). In addition to pikachurin, an extracellular matrix-like protein located in the photoreceptor synaptic cleft, we also verified that the presynaptic proteins CACNA1F and cone arrestin (mCAR), had normal immunoreactivity in the *Lrit3^-/-^* OPL (Extended Data Fig. 2-1). In contrast, we also find a dramatic decrease in PNA staining in *Lrit3^-/-^*, consistent with previous observations (Extended Data Fig. 2-1; Neuillé et al., 2015). These data show that the absence of LRIT3 not only impacts the postsynaptic ON bipolar signalplex, but also alters expression of at least one presynaptic protein. *Lrit3^-/-^*RGC light evoked signaling is abnormal in other “no b-wave” mutant mouse models of cCSNB, RGCs show several abnormalities in spontaneous and visually evoked activity (*Nyx^nob^* and *Grm6^-/-^*; Demas et al., 2006; Peachey et al., 2017). Abnormalities include a decrease in the proportion of cells that response to light onset (ON), as well as a significant delayed TTP in those RGCs that retain a response to light onset (dON). In addition, most RGCs have a dominant rhythmic bursting component in both their spontaneous and light evoked activity. Notably, *Nyx^nob^* and *Grm6^-/-^* RGCs retain responses to light offset (OFF) that are the same as control.

10.1523/ENEURO.0002-20.2020.f2-1 Extended Data Figure 2-1 Cone terminal appear normal in *Lrit3^-/-^*retinas. ***A***, Immunohistochemical staining for the presynaptic markers CACNA1F and PNA. CACNA1F staining in *Lrit3^-/-^* retinas is indistinguishable from controls. PNA staining in *Lrit3^-/-^* retinas is decreased, but not completely absent. ***B***, Staining for the cone terminal marker mCAR (cone arrestin) and PNA. mCAR staining in the *Lrit3^-/-^* retinas is similar to controls, and PNA is decreased in *Lrit3^-/-^* retinas. Download Figure 2-1, TIF file.

**Figure 3.**
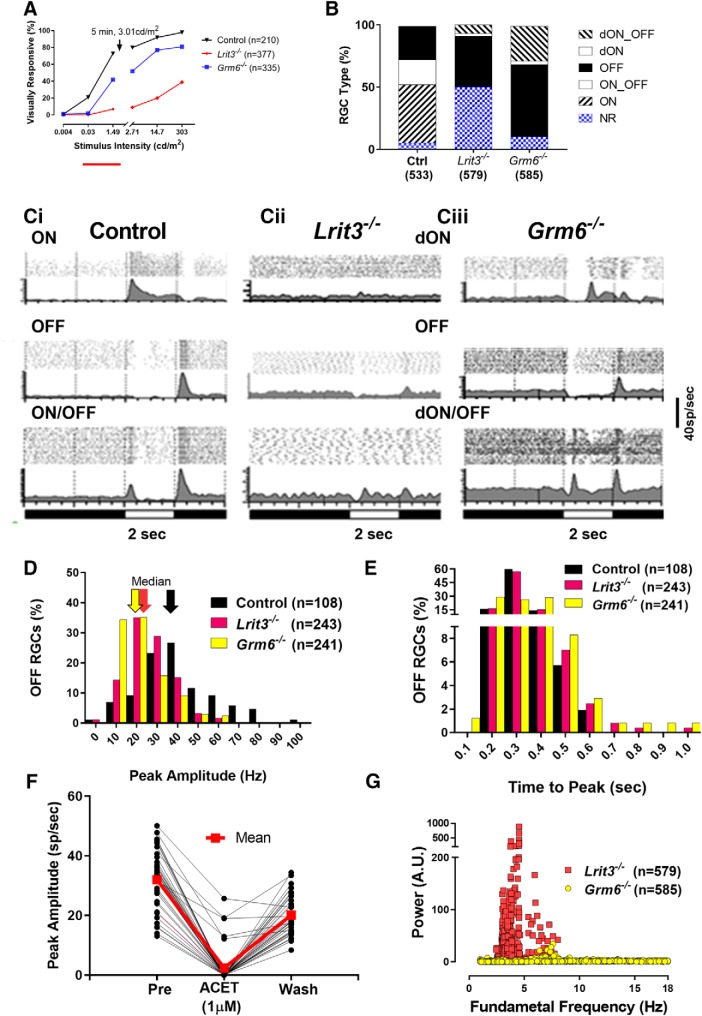
Visual responses of *Lrit3^-/-^*RGCs are significantly altered compared to controls and *Grm6^-/-^*. ***A***, Intensity response functions of control, *Lrit3^-/-^*, and *Grm6^-/-^* RGCs. Break in the *x*-axis represents 5-min light adaptation to a background of 3.01 cd/m^2^. ***B***, RGC functional classes in control, *Lrit3^-/-^* and *Grm6^-/-^* retinas based on responses to a 303 cd/m^2^ full field flashes on a 0.3 cd/m^2^ background. The total number of RGCs for each genotype is shown on the *x*-axis and were obtained from 12 retinal pieces from eight control mice, 16 retinal pieces from 6 *Lrit3^-/-^* mice, and 10 retinal pieces from four *Grm6^-/-^* mice, respectively. There are significantly more visually non-responsive RGCs (defined as cells with spontaneous but no visually evoked activity) in the *Lrti3^-/-^* mice than either control or *Grm6^-^/-*(∼50% increase; Fishers exact test, *p* < 0.001 for both comparisons). ***C***, Representative average PSTHs (above, raster plots to individual stimulus presentation) of responses to a full field light stimulus (303 cd/m^2^) on a 0.3 cd/m^2^ background, recorded on a MEA. ***Ci***, Control RGC responses can be classified as ON, OFF, and ON/OFF based on whether spiking peaks to light onset, offset or both, respectively. All responses occur <0.4 s after stimulus onset. *Lrit3^-/-^* (***Cii***) and *Grm6^-/-^* (***Ciii***) RGC responses can be classified into the same general groups but the time to the peak response to light onset is >0.4 s (summary data not shown), and these responses are referred to as delayed ON (dON) or dON/OFF RGCs. ***D***, The peak amplitude of OFF RGCs in *Lrit3^-/-^* and *Grm6^-/-^* are decreased compared to controls (median: 25 and 19 Hz, respectively, vs 38 Hz in controls, Kruskal–Wallis followed by Dunn’s test; *p* < 0.001 for both comparisons). ***E***, Distribution of response latencies in the OFF *Lrit3^-/-^* and *Grm6^-/-^* RGCs is not different from control (Kruskal–Wallis *p* = 0.995 for both comparisons). ***F***, OFF responses in *Lrit3^-/-^* RGCs are mediated by kainate receptors. Responses show 37 RGCs peak response (sp/s), before (32 ± 1.6 SEM), during (2.4 ± 1.1), and after ACET (20.11 ± 1.1; 1.0 μM) treatment. ***G***, Fast Fourier transform to quantify rhythmicity in the spontaneous activity of *Lrit3^-/-^* and *Grm6^-/-^* RGCs. *Lrit3^-/-^* cells exhibit rhythmic firing, whereas few do so in the *Grm6^-/-^* retinas.

We surveyed the responses of ∼1000 RGCs in *Lrit3^-/-^*, *Grm6^-/-^* and control retinas using a MEA. To optimize stimulus conditions, we first evaluated responses in a subset of these RGCs to flashes at several luminance intensities in dark-adapted and in light-adapted retinal whole-mounts (Fig. 3*A*). The dimmest scotopic flash (0.004 cd/m^2^) evoked responses in a small proportion of RGCs across these three genotypes. The next brightest scotopic flash (0.03 cd/m^2^) evoked responses in significantly fewer *Grm6^-/-^* and *Lrit3^-/-^* RGCs compared to control. All of the other scotopic and photopic stimuli evoked responses in significantly fewer *Grm6^-/-^* and *Lrit3^-/-^* RGCs compared to control (*p* < 0.05, Fisher exact tests with Bonferroni correction). We also found a large and unexpected difference in the percentage of *Lrit3^-/-^* versus *Grm6^-/-^* NR RGCs, e.g., there were significantly more NR *Lrit3^-/-^* versus *Grm6^-/-^* RGCs (50% and 10%, respectively; Fishers *p* < 0.0001). The observed differences in the proportions of NR RGCs prompted us to examine other aspects of the *Lrit3^-/-^* mouse visually evoked responses. Using the brightest photopic flash stimulus (303 cd/m^2^), we subdivided the visually responsive RGCs in each of the three genotypes (control, *Lrit3^-/-^* and *Grm6^-/-^*) into three functional groups (Fig. 3*C*): (1) RGCs with responses to light onset (ON, top row), which could be subdivided into ON or delayed ON (dON, TTP > 500 ms); (2) RGCs with a response to light offset (OFF, middle row); and (4) RGCs with a response to light onset and offset (ON/OFF, bottom row). Among the visually responsive control RGCs, 47% increased spiking at light onset; 27% increased spiking at light offset; and 20% increased spiking to both luminance changes. Against this baseline, we compared responses in *Lrit3* ^-/-^ and *Grm6^-/-^* RGCs, which share a no b-wave phenotype. The mutant RGC response types differed strikingly from control (Fig. 3*B*). As expected from a no b-wave phenotype, neither *Lrit3^-/-^* nor *Grm6^-/-^* RGCs showed short latency ON responses, although both had RGC ON responses with significantly delayed onsets compared to control (>0.5 s, TTP; 9% in *Lrit3^-/-^* vs 31% in *Grm6^-/-^* RGCs).

The large number of *Lrit3^-/-^* NR cells suggests that sensitivity of the visual response may be impacted by the loss of LRIT3 expression. To examine this, we compared the peak response of RGCs across genotype. Peak responses in *Lrit3^-/-^* OFF RGCs were significantly smaller than control (median = 24.7 sp/s, *n* = 242 vs median = 37.6 sp/s, *n* = 86), although they were larger than *Grm6* ^-/-^ RGCs (18.5 sp/s, *n* = 241 (Fig. 3*D*); Kruskal–Wallis, all comparisons *p* < 0.0001). In contrast, the TTP response of OFF RGCs was similar across genotypes (Fig. 3*E*; Kruskal–Wallis, *p* = 0.995). Among the few *Lrit3^-/-^* ON/OFF RGCs, the TTP firing at light onset (median = 660 ms, *n* = 8) was significantly delayed compared to control (median = 250 ms, *n* = 263) and was similar to *Grm6^-/-^* RGCs (median = 550 ms, *n* = 35; Peachey et al., 2017).

Since crossover input from the ON pathway is absent, we could use the kainate receptor antagonist ACET (1 μM; Fig. 3*F*) to verify that the *Lrit3^-/-^* OFF RGCs responses were in fact initiated in the OFF signaling pathway. We compared light evoked responses in control bath solution to the same RGCs in the presence of ACET. Of 515 *Lrit3^-/-^* RGCs, 180 (35%) had visually evoked OFF responses in control solution and only 11 (6%) retained that response after addition of ACET to the bath. Further, all OFF RGCs showed a reduction in peak response amplitude (∼43% of control). After 1-h wash, 37 RGCs recovered their OFF response. These data demonstrate that light evoked *Lrit3^-/-^* OFF responses.

*Nyx^nob^* RGCs show rhythmic bursting in their spontaneous activity, which also is superimposed on their light evoked responses (Demas et al., 2006). This activity is absent from *Grm6^-/-^* RGCs (Rentería et al., 2006). Both the poststimulus time histograms and the raster plots (Fig. 3*Cii*) suggest that rhythmic activity is present in *Lrit3^-/-^* RGCs and a fast Fourier transform was used to extract this activity and we found *Lrit3^-/-^* RGCs have a frequency between 3 and 8 Hz, similar to *Nyx^nob^* RGCs (Fig. 3*G*) and the rhythmicity is present in visually responsive and NR *Lrit3^-/-^* RGCs. Under the same recording conditions, the few *Grm6^-/-^* RGCs that did have rhythmic activity (Fig. 3*G*; 24/585) had fundamental frequencies between 5.6 and 9 Hz, but the modulation amplitude was ∼3-fold lower compared with *Lrit3^-/-^* RGCs (Fig. 3*G*).

### *Lrit3^-/-^* inner plexiform layer (IPL) shows no on and reduced off glutamate release

**Figure 4.**
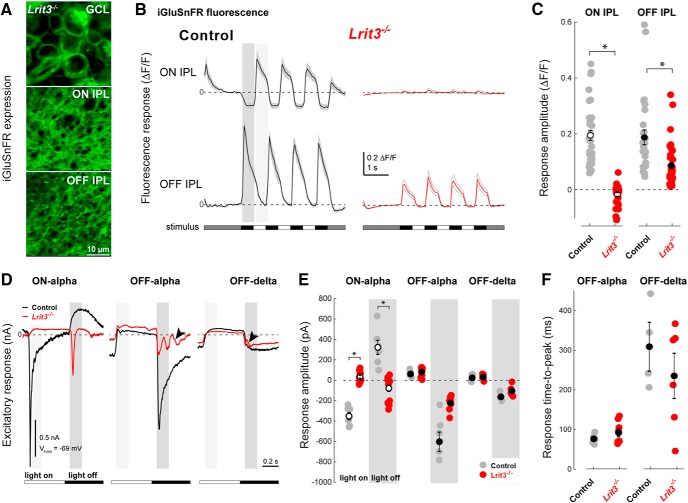
Glutamate release in *Lrit3^-/-^*mice is decreased in both ON and OFF sublaminae of the IPL. ***A***, iGluSnFR expression in somas, and dendrites in the ON and OFF sublaminae of the IPL following viral transduction with AAV2/1-*hsyn*-iGluSnFR. ***B***, Visually-evoked changes in glutamate levels as detected by changes in iGluSnFR fluorescence in the ON (top traces) and OFF (bottom traces) layers of the IPL for control (black) and *Lrit3^-/-^* (red) retinas, to a contrast reversing spot on a photopic background (1.2 × 10^4^ R*/rod and cone/s; 150 μm in diameter, 1-Hz square wave, 100% Michelson contrast). ***C***, Summary data for the change in iGluSnFR fluorescence in the ON and OFF sublaminae of control and *Lrti3^-/-^* retinas for all recorded areas (ON, control *n* = 37, seven eyes; *Lrit3^-/-^ n* = 35, eight eyes; OFF, control *n* = 28, seven eyes; *Lrit3^-/-^ n* = 40, eight eyes). ***D***, Whole-cell electrophysiological recordings of light-evoked current responses of three identified RGC types (same stimulus as in ***B***, except diameter 350 μm; recorded in voltage clamp mode at the reversal potential for chloride, –69 mV). ***E***, ***F***, Summary data for the light-evoked current response amplitude for all recorded cells. ON and OFF response amplitudes (***E***) were computed as the mean of the recorded current following a light increment and decrement, respectively (light and dark gray regions in ***D***). For all responses the amplitude of the excitatory (inward) current is inverted for ease of interpretation. Response amplitudes from control versus *Lrit3^-/-^* were compared using a *t* test (ON α ON response, control *n* = 7, five eyes vs *Lrit3^-/-^ n* = 15, eight eyes, *p* < 0.0001; OFF α OFF response; control *n* = 7, five eyes vs *Lrit3^-/-^ n* = 8, six eyes, *p* < 0.0007; OFF δ OFF response control *n* = 4, four eyes, *Lrit3^-/-^ n* = 6, four eyes, *p* = 0.086). TTP of the OFF responses of OFF-α and OFF-δ ganglion cells (***F***) were defined as the time following onset of a light decrement (dark gray region in ***D***) when the excitatory current peaked. TTP of control versus *Lrit3^-/-^* were compared using a *t* test (OFF α OFF response; control *n* = 7, five eyes vs *Lrit3^-/-^ n* = 8, six eyes, *p* = 0.183; OFF δ OFF response control *n* = 4, four eyes, *Lrit3^-/-^ n* = 6, four eyes, *p* = 0.0.371). All error bars are mean ± SEM. * = Significant difference.

The large number of *Lrit3^-/-^* OFF RGCs with reduced peak firing rates led us to examine light-evoked glutamate release from BC axon terminals in the IPL sublaminae. The fluorescent glutamate sensor iGluSnFR was expressed in RGCs and amacrine cells using viral transduction, and changes in iGluSnFR fluorescence in the middle of the ON and OFF sublaminae were imaged during visual stimulation using two-photon fluorescence microscopy (Fig. 4*A*; Borghuis et al., 2013). At light onset and offset, control retinas showed robust fluorescence responses in both the ON (ON responses) and OFF IPL sublaminae (OFF responses), reflecting glutamate release from ON and OFF-BCs, respectively (Fig. 4*B*). In the *Lrit3^-/-^* IPL, glutamate release was negligible in the ON sublamina compared to control (–0.017 ± 0.006, *n* = 35; eight eyes vs 0.194 ± 0.017, *n* = 37; seven eyes; *t* test, *p* < 0.0001; Fig. 4*B*). Although we observed unambiguous stimulus modulated glutamate release in the *Lrit3^-/-^* OFF sublamina, following light decrements, the amplitude of these responses (ΔF/F) was reduced compared with control (0.086 ± 0.012, *n* = 40; eight eyes vs 0.187 ± 0.026, *n* = 28; seven eyes; *t* test, *p* = 0.0002; Fig. 4*B*,*C*), which is consistent with our MEA results.

To understand the changes underlying the reduced light signaling in ON and OFF pathways, we compared control and *Lrit3^-/-^* ON α, OFF α, and OFF δ RGCs using whole-cell voltage clamp recordings in whole-mount retina preparations (Fig. 4*D*). RGC identity was confirmed based on dendritic morphology from fills with sulforhodamine 101, included in the intracellular pipette solution.

Clamped at the reversal potential for chloride (ECl; –69 mV), control ON α RGCs showed a robust inward (excitatory) current at light onset (ON response) and suppression of tonic inward current at light offset (OFF response), reflecting the stimulus-evoked modulation of glutamate release from presynaptic ON BCs. In *Lrit3^-/-^* ON α RGCs, the inward current at light onset was lost compared to control (33.4 ± 12.6 pA, *n* = 15; eight eyes vs –352.1 ± 32.4 pA, *n* = 7; five eyes; *t* test, *p* < 0.0001; Fig. 4*E*). In addition, in *Lrit3^-/-^* ON α RGCs the suppression following light offset was replaced by a small transient inward current in the *Lrit3^-/-^* cells (–78.4 ± 30.9 pA, *n* = 15; eight eyes vs 322.6 ± 64.1 pA, *n* = 7; five eyes; *t* test, *p* < 0.0001). The emergence of an excitatory OFF response in ON α GCs is likely to result from AII amacrine-cell mediated signaling from OFF BCs to ON BCs, but was not investigated further. Clamped at the reversal potential for chloride (ECl; –69 mV), control OFF α and OFF δ RGCs showed inward currents at light offset, as expected (Borghuis et al., 2014). Although the response polarity and timing in *Lrit3^-/-^* OFF α RGCs were similar to control, response amplitude was reduced significantly (–222.8 ± 25.7 pA, *n* = 8; six eyes vs –603.2 ± 87.4 pA, *n* = 7; five eyes; *t* test, *p* = 0.0007), whereas in the OFF δ RGCs responses in *Lrit3^-/-^* and control OFF were similar (–104.5 ± 20.4 pA, *n* = 6; four eyes vs –163.1 ± 20.0 pA, *n* = 4; four eyes; *t* test, *p* = 0.086; Fig. 4*E*). Also note that there are oscillations in the response in the *Lrit3^-/-^* OFF α and δ RGCs (Fig. 4*D*, arrowheads) that are absent from control RGCs.

In sum, our electrophysiological recordings show that *Lrit3^-/-^* ON α RGCs lack excitatory synaptic input at light onset, as expected from their no b-wave phenotype and the absence of ON RGC spiking responses. What is novel about this no b-wave model is that the amplitudes of light-evoked. excitatory currents in at least one identified OFF RGC type are reduced, consistent with a decrease in their spiking activity. Collectively, the MEA and voltage clamp data suggest that light-evoked responses of many *Lrit3^-/-^* OFF RGCs are less sensitive to light compared to control RGCs, although the magnitude of the impact may vary with OFF RGC type. Given that LRIT3 is not expressed in the IPL (Extended Data Fig. 1-1*D*), it is possible that *Lrit3^-/-^* OFF RGCs have reduced synaptic input from the presynaptic OFF BCs.

### ON and OFF *Lrit3^-/-^*BC function is abnormal

**Figure 5.**
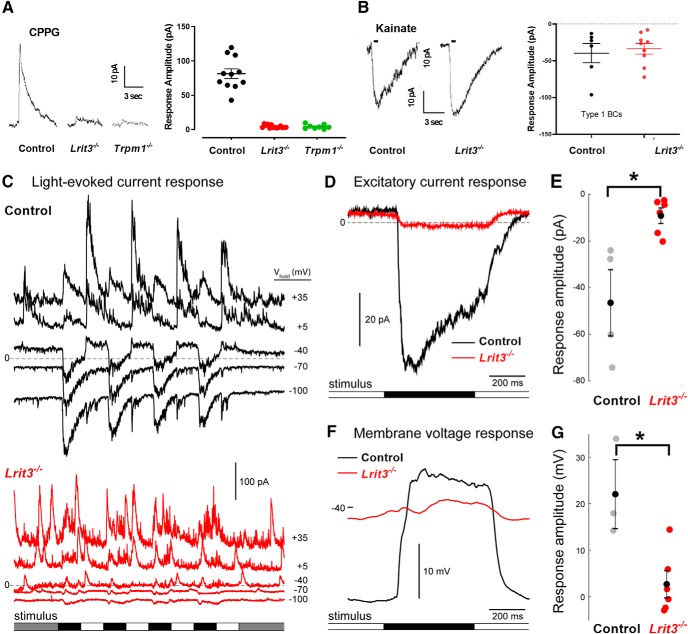
*Lrit3^-/-^*rod BCs lack functional Trpm1 responses and BC1 cells have decreased excitatory input. ***A***, Whole-cell voltage clamp recordings from rod BC from control, *Lrit3^-/-^* and *Trpm1^-/-^* mice during puffs of the mGluR6 antagonist CPPG (3 mM). Summary data shows a decreased response in *Lrit3^-/-^* (*n* = 11) and *Trpm1^-/-^* (*n* = 11) cells compared to control, consistent with absence of the ERG b-wave in these knock-outs (***ANOVA, *F*_(2,30)_, *p* < 0.001, Tukey *post hoc* tests, *p* < 0.001 for control vs both knock-outs). ***B***, Responses from BC1 cells to kainate puffs (50 μm) in retinal slices from control and *Lrit3^-/-^* retinas. Summary data for all recorded cells shows no difference in the maximal responses between the two genotypes (*t* test, *p* = 0.67). ***C***, Whole-cell voltage clamp recordings of BC1 cell in control (top) and *Lrit3^-/-^* (bottom) mouse whole-mounts. Traces represent the current recorded at different holding potentials (ECl = –69 mV; Ecat = 0 mV). *Lrit3^-/-^* showed a marked absence of excitatory current (lower traces). ***D***, Light-evoked excitatory current response of an example control and *Lrit3^-/-^* BC1 cell. Traces show the average response to nine repeats of the square wave, contrast-reversing spot stimulus presented on a photopic background (1.2 × 10^4^ R*/rod and cone/s; 150 μm in diameter, 1-Hz square wave, 100% Michelson contrast). ***E***, Summary data for the excitatory current response for all recorded BC1 cells (data partially shown in ***D***) control *n* = 4, three eyes; *Lrit3^-/-^ n* = 6, four eyes; *t* test *p* = 0.0072). ***F***, ***G***, As for ***D***, ***E***, for membrane voltage response recorded in current clamp mode (control *n* = 6, four eyes; *Lrit3^-/-^ n* = 3, three eyes; *t* test *p* = 0.0105). Error bars are mean ± SEM. * = Significant difference.

To determine whether ON pathway excitatory input to ON α RGCs is eliminated, we assessed TRPM1 mediated currents in *Lrit3^-/-^* ON rod BCs, using whole cell recordings (Fig. 5*A*). We simulated dark by bathing retinal slices in L-APB to activate mGluR6 and close TRPM1, then inactivated the mGluR6 to TRPM1 cascade by puffing on CPPG to gate TRPM1 (Shen et al., 2009). In control rod BCs, CPPG mediated a robust current (81.5 ± 7.1 pA, *n* = 11) that was absent in both *Trpm1^-/-^* (4.4 ± 0.6 pA, *n* = 8) and *Lrit3^-/-^* (4.2 ± 1.1 pA, *n* = 14) rod BCs (Fig. 5*A*). The small residual current in both knock-outs has been observed previously, although its source remains unknown (Ray et al., 2014). We assessed excitatory input to *Lrit3^-/-^* OFF BCs, to determine whether it underlies the decreased glutamate release in the OFF sublamina. We first assessed AMPA/kainate receptor mediated currents in *Lrit3^-/-^* type 1 fluorescent OFF BCs. Kainate puffs evoked robust inward currents (Fig. 5*B*) and the response amplitudes in control and *Lrit3^-/-^* BCs were similar (–39.7 ± 13.3 pA, *n* = 6 vs –33.7 ± 7.2 pA, *n* = 9, *t* test *p* = 0.67). Thus, the absence of LRIT3 eliminates mGluR6 mediated responses in DBCs, but does not impact the kainate mediated input to OFF BC1s.

We examined the light evoked responses of BC1s using two-photon whole-cell recording in a whole-mount retinal preparation. Control BC1 responses to light stimuli showed robust excitatory and inhibitory currents, over a range of holding potentials (V_hold_; Fig. 5*C*). The control response was characterized by an excitatory current to light decrements recorded at ECl (∼–60 mV) and an inhibitory current at Ecation (∼0 mV) to both light decrements and increments. This is consistent with the view that BC1s receive feed-forward excitation (OFF pathway-mediated), a combination of crossover ON inhibition likely mediated by ON pathway input from AII amacrine cells, and feed-forward inhibition via the OFF pathway. Excitatory currents in *Lrit3^-/-^* BC1s at light offset (OFF pathway input) were dramatically reduced compared to control (–9.1 ± 3.0 pA, *n* = 6; four eyes vs –46.5 ± 12.2 pA, *n* = 4; three eyes; *t* test, *p* = 0.0072), and the initial transient component was absent (Fig. 5*D*,*E*). Membrane voltage responses recorded in current clamp mode showed substantial depolarization at light offset in control BC1s, which is absent in *Lrit3^-/-^* BC1s (22.0 ± 6.1 pA, *n* = 3; three eyes vs 2.7 ± 2.7 pA, *n* = 6; five eyes; *t* test, *p* = 0.0105; Fig. 5*F*). Finally, the inhibitory currents in *Lrit3^-/-^* BC1s are desynchronized to the stimulus.

Taken together, we observe reduced excitatory responses in BC1s, although their response to exogenous kainate application is unaffected. In addition, glutamate release in *Lrit3^-/-^* IPL OFF sublamina (Fig. 4*B*), excitatory signaling to OFF *Lrit3^-/-^* α RGCs and the majority of OFF RGC spiking responses are reduced. Combined the data strongly support the idea that LRIT3 is important for glutamate dynamics and/or sensing by OFF BCs in the synaptic cleft.

## Discussion

In this report, we confirm and expand on the nature of the previously reported deficits in the ON pathway in the absence of LRIT3 (Ray, 2013; Neuillé et al., 2014; Hasan et al., 2019). We now show that OFF pathway function also is severely impacted by the absence of LRIT3. The significant reduction in OFF BC function leads to abnormal OFF RGC excitatory inputs and spiking responses, which has not been reported either in *Lrit3^nob6^* or in two other CSNB1 mutant mouse models, *Nyx^nob^* and *Grm6^-/-^* (Demas et al., 2006; Maddox et al., 2008).

Mutations in *LRIT3* were first identified from DNA samples of CSNB1 patients, using exome sequencing (Zeitz et al., 2013). ERG characterization of *Lrit3^-/-^* mouse retina (Extended Data Fig. 1-1; Ray, 2013; Neuillé et al., 2014) show a typical no ERG b-wave phenotype, similar to several other CSNB1 mouse models (Masu et al., 1995; Pardue et al., 1998; Morgans et al., 2009; Shen et al., 2009; Koike et al., 2010; Peachey et al., 2012). This phenotype is consistent with the expression pattern of LRIT3 in the mouse retina, where it colocalizes with key DBC signalplex proteins, including nyctalopin, TRPM1, mGluR6, GPR179, nyctalopin, RGS7, RGS11, Gβ5, and R9AP (Fig. 1; Extended Data Figs. 1-1, 1-2; Ray, 2013; Neuillé et al., 2015, 2017; Hasan et al., 2019). The absence of LRIT3 expression has a negative impact on TRPM1 localization on rod DBC dendrites (Extended Data Fig. 1-1; Neuillé et al., 2015; Hasan et al., 2019). From this result Neuillé et al. (2015) concluded that TRPM1 expression depended on LRIT3 at the DBC synapse. We now extend this observation and our current results with previous results (Pearring et al., 2011) and suggest that the loss of TRPM1 is likely secondary to the loss of nyctalopin from the DBC dendritic tips in *Lrit3^-/-^* mice (Fig. 1), which is required for TRPM1 localization (Pearring et al., 2011).

Further, our data suggest that nyctalopin insertion is likely because of a transsynaptic interaction between LRIT3 and nyctalopin (Fig. 1; Hasan et al., 2019), which establishes a scaffold for assembly of TRPM1.

While nyctalopin and thus TRPM1, are absent from the tips of all DBCs, the situation with respect to the remaining DBC signalplex components is more complex. At the *Lrit3^-/-^* rod BCs dendritic tips, mGluR6, GPR179, RGS7, RGS11 and R9AP are all present, whereas they are absent from the cone DBC dendrites (Extended Data Fig. 1-2; Ray, 2013; Neuillé et al., 2015). Further, the absence of LRIT3 decreases, but does not eliminate, the PNA staining on cone terminals (Extended Data Fig. 2-1; Neuillé et al., 2015). LRIT3 is glycosylated, but the molecular composition of that glycosylation and whether PNA binds it is unknown. Our data suggest that LRIT3 has more complex interactions at the cone to cone DBCs than at the rod synapse. Given the impact of the absence of LRIT3 on light-driven input to the type 1 BCs (see below), it is possible LRIT3 disrupts aspects of cone synaptic function.

### LRIT3 is required for both on and off pathway signaling in RGCs

The light-evoked *Lrit3^-/-^* RGCs responses to light onset are similar to RGCs responses characterized in several CSNB1 mouse lines *Nyx^nob^* (Demas et al., 2006), *Grm6^-/-^* (Rentería et al., 2006; Pinto et al., 2007; Peachey et al., 2017; Fig. 3), *Trpm1^-/-^* (Takeuchi et al., 2018), and *Lrit3^-/-^* (Fig. 3) and *Lrit3^nob6^* (Neuillé et al., 2017). *Lrit3^-/-^* mice, like most of these models show an increase in RGCs with spontaneous and no light evoked responses, most have no RGCs with ON responses comparable to control and the only ON responses have significantly delayed TTP. In *Grm6^-/-^* retina this input has been shown to arise from OFF BC input, via crossover signaling (Rentería et al., 2006). Finally, like *Nyx^nob^* and *Trpm1^-/-^*, *Lrit3^-/-^* RGCs show a rhythmic bursting component in their spontaneous activity.

The defects in the OFF pathway are unique to the *Lrit3^-/-^* RGCs. The MEA surveys a large population of RGCs simultaneously, and we found that the peak amplitude of the OFF response is decreased compared to control OFF RGCs. Consistent with this change in the spiking response the excitatory currents in the OFF α RGCs also are reduced. Further, there is a reduction in the glutamate released in the OFF sublaminae of the *Lrit3^-/-^* IPL. A consequence of these affects is that even at the brightest stimulus intensities 60% of the *Lrit3^-/-^* RGCs are visually non-responsive compared to 19% in *Grm6^-/-^* and 2% in control retinas. The decrease in response amplitude and increase in NR RGCs in *Lrit3^-/-^* retinas can, in part, be attributed to the lack of ON input, but the large difference in the number of NR RGCs compared to of *Grm6^-/-^* retinas suggests an additional defect in *Lrit3^-/-^* OFF pathway signaling.

Zeitz and colleagues reported that the *Lrit3^nob6^* OFF RGCs had a delayed TTP (Neuillé et al., 2017). In contrast, we find a normal TTP in *Lrit3^-/-^* OFF RGCs measured using the MEA or by patch clamp recordings. The difference they observe arises from the distribution of their WT response latencies in their small dataset, which all are very fast. In contrast, while our larger dataset contains *Lrit3^-/-^* OFF RGCs with fast TTP, the distribution is broader and is not different from controls. Given the difference in TTP of OFF α (fast) and δ (slow) RGC excitatory currents (Fig. 4*E*), it is possible that Neuillé and colleagues control dataset represent a different subpopulation than we record.

The interpretation of our recordings of excitatory currents in OFF α and OFF δ RGCs suggest signaling through the OFF pathway in *Lrit3^-/-^* retinas is complex. *Lrit3^-/-^* OFF α cells show a large decrease in light-evoked excitatory current compared to control, whereas the *Lrit3^-/-^* OFF δ cells are similar to controls. Of particular note is that the decrease in *Lrit3^-/-^* OFF-α cells (∼90%) is far greater than the decrease observed in control OFF-α cells recorded in the presence of L-AP4 (∼25% decrease in excitatory current (Borghuis et al., 2014). Furthermore, when control OFF δ cells are recorded in the presence of L-AP4, their excitatory currents actually increase (Borghuis et al., 2014). Based on these data we postulate that the different effects of the absence of LRIT3 on the OFF α and δ RGCs results from a direct decrease in excitatory input from OFF BCs combined with alterations in crossover inputs that differ between the types of RGC.

### The role of LRIT3 in off pathway signaling in HBCs

The deficits in OFF RGC signaling, led us to examine potential dysfunction of *Lrit3^-/-^* OFF BCs and their excitatory inputs to OFF RGCs. We find that the kainate receptor mediated input to *Lrit3^-/-^* and control type 1 OFF BCs is similar, whereas the light evoked input to the *Lrit3^-/-^* BCs is significantly smaller than control. The decrease in glutamate release in the IPL and the decreased amplitude of OFF RGCs is consistent with a general decrease in HBC excitatory input. While the mechanism underlying the decreased excitatory input to OFF BCs requires further study, it is now clear that it does not reflect a defect in photoreceptor visual transduction because the *Lrit3^-/-^* scotopic and photopic a- waves are similar to controls (Extended Data Fig. 1-1). Thus, the first stage of altered signaling appears to be the glutamatergic transmission at the photoreceptor→BC synapse. This defect could arise from either structural alterations at the synapse or alterations in glutamate dynamics. Electron microscopy studies of the OPL from *Lrit3^nob6^* retinas indicate that there may be minor alterations to the invaginating synapse, although flat contacts were reportedly normal (Neuillé et al., 2017). Immunohistochemical studies of cone markers CACNA1F and mCAR also did not reveal any alterations in *Lrit3^-/-^* retinas (Extended Data Fig. 2-1). Because of the decrease in excitatory input to type 1 BCs we wondered if there were changes in glutamate release or uptake from the synapse. To examine this we used iGluSnFR expression in Mueller cells to measure glutamate dynamics in the OPL (Borghuis et al., 2013). We were unable to demonstrate any changes in the glutamate dynamics in the *Lrit3^-/-^* retinas with this method (unpublished data), although the challenging nature of these experiments precludes detection of subtle changes.

Finally, the fact that LRIT3 is expressed by rods (Hasan et al., 2019), suggests this could be the case for cones and combined with the extensive loss of cone DBC signalplex components, it is possible that the absence of LRIT3 may also alter additional components critical to synaptic function. These issues remain to be elucidated in future studies.

### Summary

Our studies show that LRIT3 controls the localization of nyctalopin to the DBC dendritic tips, and that LRIT3 is crucial for normal excitatory input to OFF BCs. We propose that the dependence of the OFF pathway function on LRIT3 expression results from a dual role of LRIT3. First, it is required for normal assembly of the DBC signalplex, and second, for synaptic glutamate dynamics that decrease input to OFF BCs, and thus decreased excitatory responses in their postsynaptic OFF RGC targets.

## Synthesis

Reviewing Editor: Kirill Martemyanov, The Scripps Research Institute Decisions are customarily a result of the Reviewing Editor and the peer reviewers coming together and discussing their recommendations until a consensus is reached. When revisions are invited, a fact-based synthesis statement explaining their decision and outlining what is needed to prepare a revision will be listed below. Note: If this manuscript was transferred from JNeurosci and a decision was made to accept the manuscript without peer review, a brief statement to this effect will instead be what is listed below.

The manuscript was accepted after editorial review.

## References

[B1] Ball SL, Powers PA, Shin HS, Morgans CW, Peachey NS, Gregg RG (2002) Role of the β2 subunit of voltage-dependent calcium channels in the retinal outer plexiform layer. Invest Ophthalmol Vis Sci 43:1595–1603. 11980879

[B2] Ball SL, Pardue M, McCall M, Gregg R, Peachey N (2003) Immunohistochemical analysis of the outer plexiform layer in the Nob mouse. Invest Ophthalmol Vis Sci 44:1865. 10.1017/s0952523803203059 14570248

[B3] Borghuis BG, Marvin JS, Looger LL, Demb JB (2013) Two-photon imaging of nonlinear glutamate release dynamics at bipolar cell synapses in the mouse retina. J Neurosci 33:10972–10985. 10.1523/JNEUROSCI.1241-13.2013 23825403 PMC3718381

[B4] Borghuis BG, Looger LL, Tomita S, Demb JB (2014) Kainate receptors mediate signaling in both transient and sustained OFF bipolar cell pathways in mouse retina. J Neurosci 34:6128–6139. 10.1523/JNEUROSCI.4941-13.2014 24790183 PMC4004803

[B5] Cao Y, Posokhova E, Martemyanov KA (2011) TRPM1 forms complexes with nyctalopin in vivo and accumulates in postsynaptic compartment of ON-bipolar neurons in mGluR6- dependent manner. J Neurosci 31:11521–11526. 10.1523/JNEUROSCI.1682-11.2011 21832182 PMC3164511

[B6] Chang B, Heckenlively JR, Bayley PR, Brecha NC, Davisson MT, Hawes NL, Hirano AA, Hurd RE, Ikeda A, Johnson BA, McCall MA, Morgans CW, Nusinowitz S, Peachey NS, Rice DS, Vessey KA, Gregg RG (2006) The nob2 mouse, a null mutation in Cacna1f: anatomical and functional abnormalities in the outer retina and their consequences on ganglion cell visual responses. Vis Neurosci 23:11–24. 10.1017/S095252380623102X 16597347 PMC2831086

[B7] Demas J, Sagdullaev BT, Green E, Jaubert-Miazza L, McCall MA, Gregg RG, Wong RO, Guido W (2006) Failure to maintain eye-specific segregation in nob, a mutant with abnormally patterned retinal activity. Neuron 50:247–259. 10.1016/j.neuron.2006.03.033 16630836

[B8] DeVries SH, Schwartz EA (1999) Kainate receptors mediate synaptic transmission between cones and ‘Off’ bipolar cells in a mammalian retina. Nature 397:157–160. 10.1038/16462 9923677

[B9] Ghosh KK, Bujan S, Haverkamp S, Feigenspan A, Wässle H (2004) Types of bipolar cells in the mouse retina. J Comp Neurol 469:70–82. 10.1002/cne.10985 14689473

[B10] Gregg R, Lukasiewicz P, Peachey N, Sagdullaev B, McCall M (2003) Nyctalopin is required for signaling through depolarizing bipolar cells in the murine retina. Invest Ophthalmol Vis Sci 44:4180.

[B11] Gregg R, McCall M, Peachey N (2005) Bipolar specific expression of nyctalopin fusion gene rescues No-B wave phenotype in Nob mice. Invest Ophthalmol Vis Sci 46:3554.

[B12] Gregg RG, Ray TA, Hasan N, McCall MA, Peachey NS (2014) Interdependence among members of the mGluR6 G-protein mediated signalplex of retinal depararizing bipolar cells In: G-protein signaling mechanisms in teh retina (Martemyanov KA, Sampath AP, eds), pp 67–79. New York: Springer.

[B13] Haeseleer F, Imanishi Y, Maeda T, Possin DE, Maeda A, Lee A, Rieke F, Palczewski K (2004) Essential role of Ca2+-binding protein 4, a Cav1.4 channel regulator, in photoreceptor synaptic function. Nat Neurosci 7:1079–1087. 10.1038/nn1320 15452577 PMC1352161

[B14] Hasan N, Ray TA, Gregg RG (2016) CACNA1S expression in mouse retina: novel isoforms and antibody cross reactivity with GPR179. Vis Neurosci 33:E009 10.1017/S0952523816000055 27471951 PMC6815669

[B15] Hasan N, Pangeni G, Cobb CA, Ray TA, Nettesheim ER, Ertel KJ, Lipinski DM, McCall MA, Gregg RG (2019) Presynaptic expression of LRIT3 transsynaptically organizes the postsynaptic glutamate signaling complex containing TRPM1. Cell Rep 27:3107–3116.e3. 10.1016/j.celrep.2019.05.056 31189098 PMC6628893

[B16] Hoon M, Sinha R, Okawa H, Suzuki SC, Hirano AA, Brecha N, Rieke F, Wong RO (2015) Neurotransmission plays contrasting roles in the maturation of inhibitory synapses on axons and dendrites of retinal bipolar cells. Proc Natl Acad Sci USA 112:12840–12845. 10.1073/pnas.1510483112 26420868 PMC4611619

[B17] Ichinose T, Hellmer CB (2016) Differential signalling and glutamate receptor compositions in the OFF bipolar cell types in the mouse retina. J Physiol 594:883–894. 10.1113/JP271458 26553530 PMC4753269

[B18] Kaneko A, Saito T (1983) Ionic mechanisms underlying the responses of off-center bipolar cells in the carp retina. II. Studies on responses evoked by transretinal current stimulation. J Gen Physiol 81:603–612. 10.1085/jgp.81.4.603 6854268 PMC2215585

[B19] Kim SD, Liu JL, Roscioli T, Buckley MF, Yagnik G, Boyadjiev SA, Kim J (2012) Leucine-rich repeat, immunoglobulin-like and transmembrane domain 3 (LRIT3) is a modulator of FGFR1. FEBS Lett 586:1516–1521. 10.1016/j.febslet.2012.04.010 22673519 PMC3372856

[B20] Koike C, Numata T, Ueda H, Mori Y, Furukawa T (2010) TRPM1: a vertebrate TRP channel responsible for retinal ON bipolar function. Cell Calcium 48:95–101. 10.1016/j.ceca.2010.08.004 20846719

[B21] Maddox DM, Vessey KA, Yarbrough GL, Invergo BM, Cantrell DR, Inayat S, Balannik V, Hicks WL, Hawes NL, Byers S, Smith RS, Hurd R, Howell D, Gregg RG, Chang B, Naggert JK, Troy JB, Pinto LH, Nishina PM, McCall MA (2008) Allelic variance between GRM6 mutants, Grm6nob3 and Grm6nob4 results in differences in retinal ganglion cell visual responses. J Physiol 586:4409–4424. 10.1113/jphysiol.2008.157289 18687716 PMC2614010

[B22] Mansergh F, Orton NC, Vessey JP, Lalonde MR, Stell WK, Tremblay F, Barnes S, Rancourt DE, Bech-Hansen NT (2005) Mutation of the calcium channel gene Cacna1f disrupts calcium signaling, synaptic transmission and cellular organization in mouse retina. Hum Mol Genet 14:3035–3046. 10.1093/hmg/ddi336 16155113

[B23] Masu M, Iwakabe H, Tagawa Y, Miyoshi T, Yamashita M, Fukuda Y, Sasaki H, Hiroi K, Nakamura Y, Shigemoto R, Takada M, Nakamura K, Nakao K, Katsuki M, Nakanishi S (1995) Specific deficit of the ON response in visual transmission by targeted disruption of the mGluR6 gene. Cell 80:757–765. 10.1016/0092-8674(95)90354-2 7889569

[B24] Misgeld T, Kerschensteiner M, Bareyre FM, Burgess RW, Lichtman JW (2007) Imaging axonal transport of mitochondria in vivo. Nat Methods 4:559–561. 10.1038/nmeth1055 17558414

[B25] Morgans CW, Zhang J, Jeffrey BG, Nelson SM, Burke NS, Duvoisin RM, Brown RL (2009) TRPM1 is required for the depolarizing light response in retinal ON-bipolar cells. Proc Natl Acad Sci USA 106:19174–19178. 10.1073/pnas.0908711106 19861548 PMC2776419

[B26] Nawy S (2004) Desensitization of the mGluR6 transduction current in tiger salamander On bipolar cells. J Physiol 558:137–146. 10.1113/jphysiol.2004.064980 15146044 PMC1664922

[B27] Neuillé M, Morgans CW, Cao Y, Orhan E, Michiels C, Sahel JA, Audo I, Duvoisin RM, Martemyanov KA, Zeitz C (2015) LRIT3 is essential to localize TRPM1 to the dendritic tips of depolarizing bipolar cells and may play a role in cone synapse formation. Eur J Neurosci 42:1966–1975. 10.1111/ejn.12959 25997951 PMC4733627

[B28] Neuillé M, El Shamieh S, Orhan E, Michiels C, Antonio A, Lancelot ME, Condroyer C, Bujakowska K, Poch O, Sahel JA, Audo I, Zeitz C (2014) Lrit3 deficient mouse (nob6): a novel model of complete congenital stationary night blindness (cCSNB). PLoS One 9:e90342. 10.1371/journal.pone.0090342 24598786 PMC3943948

[B29] Neuillé M, Cao Y, Caplette R, Guerrero-Given D, Thomas C, Kamasawa N, Sahel JA, Hamel CP, Audo I, Picaud S, Martemyanov KA, Zeitz C (2017) LRIT3 differentially affects connectivity and synaptic transmission of cones to ON- and OFF-bipolar cells. Invest Ophthalmol Vis Sci 58:1768–1778. 10.1167/iovs.16-20745 28334377 PMC5374884

[B30] Pardue MT, McCall MA, LaVail MM, Gregg RG, Peachey NS (1998) A naturally occurring mouse model of X-linked congenital stationary night blindness. Invest Ophthalmol Vis Sci 39:2443–2449. 9804152

[B31] Peachey NS, Ray TA, Florijn R, Rowe LB, Sjoerdsma T, Contreras-Alcantara S, Baba K, Tosini G, Pozdeyev N, Iuvone PM, Bojang P Jr, Pearring JN, Simonsz HJ, van Genderen M, Birch DG, Traboulsi EI, Dorfman A, Lopez I, Ren H, Goldberg AF, et al. (2012) GPR179 is required for depolarizing bipolar cell function and is mutated in autosomal-recessive complete congenital stationary night blindness. Am J Hum Genet 90:331–339. 10.1016/j.ajhg.2011.12.006 22325362 PMC3276656

[B32] Peachey NS, Hasan N, FitzMaurice B, Burrill S, Pangeni G, Karst SY, Reinholdt L, Berry ML, Strobel M, Gregg RG, McCall MA, Chang B (2017) A missense mutation in Grm6 reduces but does not eliminate Mglur6 expression or rod depolarizing bipolar cell function. J Neurophysiol 118:845–854. 28490646 10.1152/jn.00888.2016 PMC5539458

[B33] Pearring JN, Bojang P Jr, Shen Y, Koike C, Furukawa T, Nawy S, Gregg RG (2011) A role for nyctalopin, a small leucine-rich repeat protein, in localizing the TRP melastatin 1 channel to retinal depolarizing bipolar cell dendrites. J Neurosci 31:10060–10066. 10.1523/JNEUROSCI.1014-11.2011 21734298 PMC3139999

[B34] Pinto LH, Vitaterna MH, Shimomura K, Siepka SM, Balannik V, McDearmon EL, Omura C, Lumayag S, Invergo BM, Glawe B, Cantrell DR, Inayat S, Olvera MA, Vessey KA, McCall MA, Maddox D, Morgans CW, Young B, Pletcher MT, Mullins RF, et al. (2007) Generation, identification and functional characterization of the nob4 mutation of Grm6 in the mouse. Vis Neurosci 24:111–123. 10.1017/S0952523807070149 17430614 PMC3770726

[B35] Ray TA (2013) Constructing the rod bipolar signalplex using animal models of retinal dysfunction. PhD thesis. University of Louisville.

[B36] Ray TA, Heath KM, Hasan N, Noel JM, Samuels IS, Martemyanov KA, Peachey NS, McCall MA, Gregg RG (2014) GPR179 is required for high sensitivity of the mGluR6 signaling cascade in depolarizing bipolar cells. J Neurosci 34:6334–6343. 10.1523/JNEUROSCI.4044-13.2014 24790204 PMC4004817

[B37] Rentería RC, Tian N, Cang J, Nakanishi S, Stryker MP, Copenhagen DR (2006) Intrinsic ON responses of the retinal OFF pathway are suppressed by the ON pathway. J Neurosci 26:11857–11869. 10.1523/JNEUROSCI.1718-06.2006 17108159 PMC2553694

[B38] Saito T, Kaneko A (1983) Ionic mechanisms underlying the responses of off-center bipolar cells in the carp retina. I. Studies on responses evoked by light. J Gen Physiol 81:589–601. 10.1085/jgp.81.4.589 6854267 PMC2215582

[B39] Sarria I, Orlandi C, McCall MA, Gregg RG, Martemyanov KA (2016) Intermolecular interaction between anchoring subunits specify subcellular targeting and function of RGS proteins in retina ON-bipolar neurons. J Neurosci 36:2915–2925. 10.1523/JNEUROSCI.3833-15.2016 26961947 PMC4783495

[B40] Sato S, Omori Y, Katoh K, Kondo M, Kanagawa M, Miyata K, Funabiki K, Koyasu T, Kajimura N, Miyoshi T, Sawai H, Kobayashi K, Tani A, Toda T, Usukura J, Tano Y, Fujikado T, Furukawa T (2008) Pikachurin, a dystroglycan ligand, is essential for photoreceptor ribbon synapse formation. Nat Neurosci 11:923–931. 10.1038/nn.2160 18641643

[B41] Shen Y, Heimel JA, Kamermans M, Peachey NS, Gregg RG, Nawy S (2009) A transient receptor potential-like channel mediates synaptic transmission in rod bipolar cells. J Neurosci 29:6088–6093. 10.1523/JNEUROSCI.0132-09.2009 19439586 PMC2752970

[B42] Slaughter MM, Miller RF (1983) An excitatory amino acid antagonist blocks cone input to sign- conserving second-order retinal neurons. Science 219:1230–1232. 10.1126/science.6131536 6131536

[B43] Takeuchi H, Horie S, Moritoh S, Matsushima H, Hori T, Kimori Y, Kitano K, Tsubo Y, Tachibana M, Koike C (2018) Different activity patterns in retinal ganglion cells of TRPM1 and mGluR6 knockout mice. Biomed Res Int 2018:2963232. 10.1155/2018/2963232 29854741 PMC5964425

[B44] Wycisk KA, Budde B, Feil S, Skosyrski S, Buzzi F, Neidhardt J, Glaus E, Nürnberg P, Ruether K, Berger W (2006) Structural and functional abnormalities of retinal ribbon synapses due to Cacna2d4 mutation. Invest Ophthalmol Vis Sci 47:3523–3530. 10.1167/iovs.06-0271 16877424

[B45] Zeitz C, Jacobson SG, Hamel CP, Bujakowska K, Neuillé M, Orhan E, Zanlonghi X, Lancelot ME, Michiels C, Schwartz SB, Bocquet B; Congenital Stationary Night Blindness Consortium, Antonio A, Audier C, Letexier M, Saraiva JP, Luu TD, Sennlaub F, Nguyen H, Poch O, et al. (2013) Whole-exome sequencing identifies LRIT3 mutations as a cause of autosomal-recessive complete congenital stationary night blindness. Am J Hum Genet 92:67–75. 10.1016/j.ajhg.2012.10.023 23246293 PMC3542465

