# Supplementary figures and images for: LRIT3 is Required for Nyctalopin Expression and Normal ON and OFF Pathway Signaling in the Retina
Source: eNeuro. 2020 Feb 6;7(1):ENEURO.0002-20.2020. doi: 10.1523/ENEURO.0002-20.2020 (PMC7031853; doi:10.1523/ENEURO.0002-20.2020)

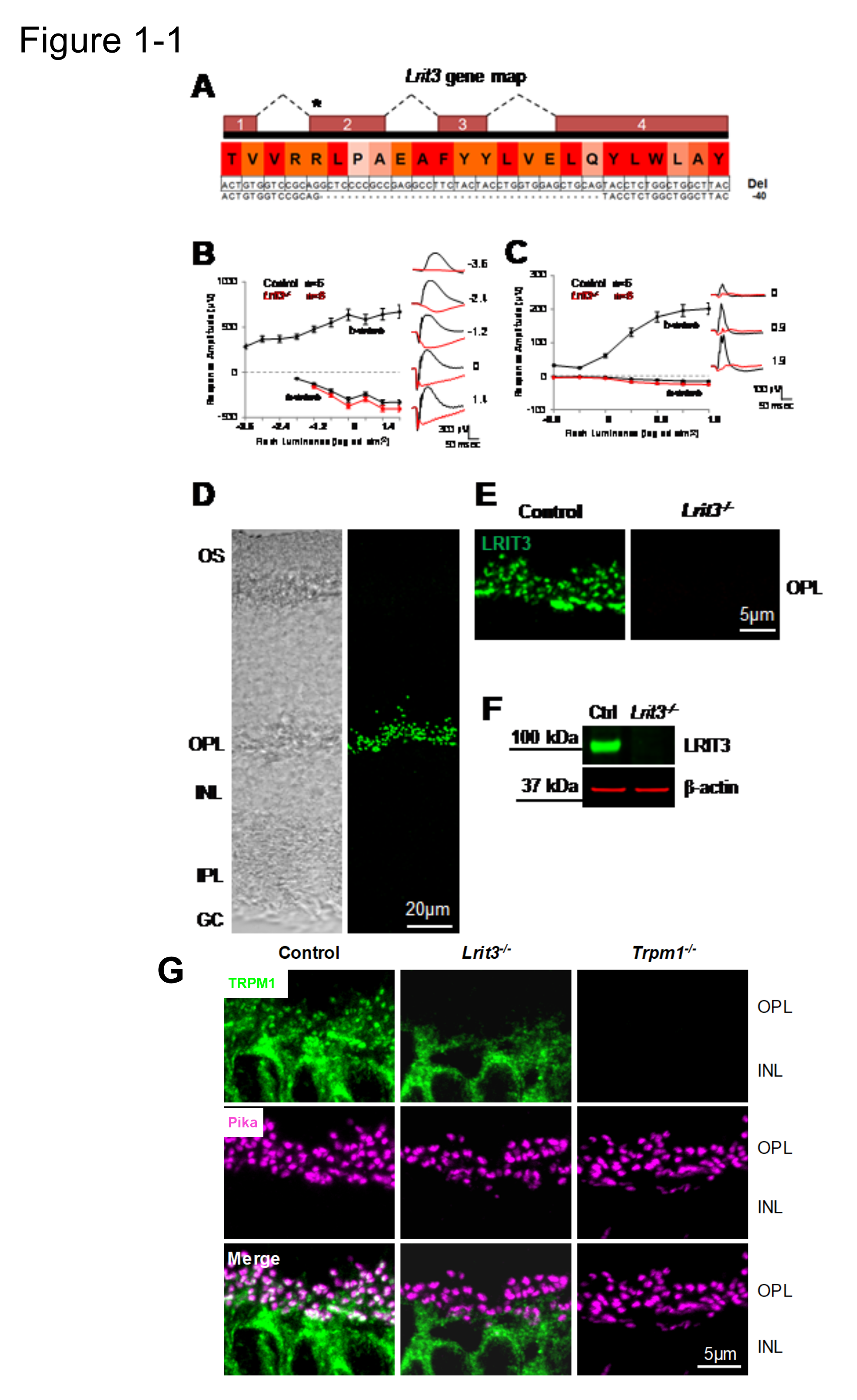

Supplement: Extended Data Figure 1-1 — LRIT3 is required for normal ERGs and is expressed in the OPL. A, Schematic of the Lrit3 gene indicating the target region (*) for the ZFN and resulting 40 bp deletion in the Lrit3-/- mouse line. B, Electroretinograms of control (black symbols) and Lrit3-/- (red symbols) mice under scotopic and (C) photopic conditions. Example response waveforms for five (scotopic) and three (photopic) luminance steps are shown, as well as summary data for all luminance steps. The Lrit3-/- mice have a normal a-wave that is similar in amplitude control mice (p > 0.05 at all flash intensities, t tests followed by Bonferroni correction for multiple testing). In contrast, Lrit3-/- mice lack the b-wave under both scotopic and photopic conditions. The control b-wave amplitude is significantly greater than 0 at all flash intensities (p > 0.05 one sample t tests followed by Bonferroni correction for multiple testing). D, DIC (left) and immunohistochemical staining for LRIT3 in transverse sections from the control mouse retina. E, LRIT3 staining of OPL of control and Lrit3-/- retinas. F, Western blotting for LRIT3 and a loading control β-actin in control and Lrit3-/- retinas. These data validate the specificity of the LRIT3 antibody is specific. G, TRPM1 (green) is mislocalized in Lrit3-/- OPL and Pikachurin expression is the same as control. These are representative images of data from at least four mice. OS, outer segments; INL, inner nuclear layer; GC, ganglion cell layer. Download Figure 1-1, TIF file. [file sup_enu-eN-NWR-0002-20-s01.tif]

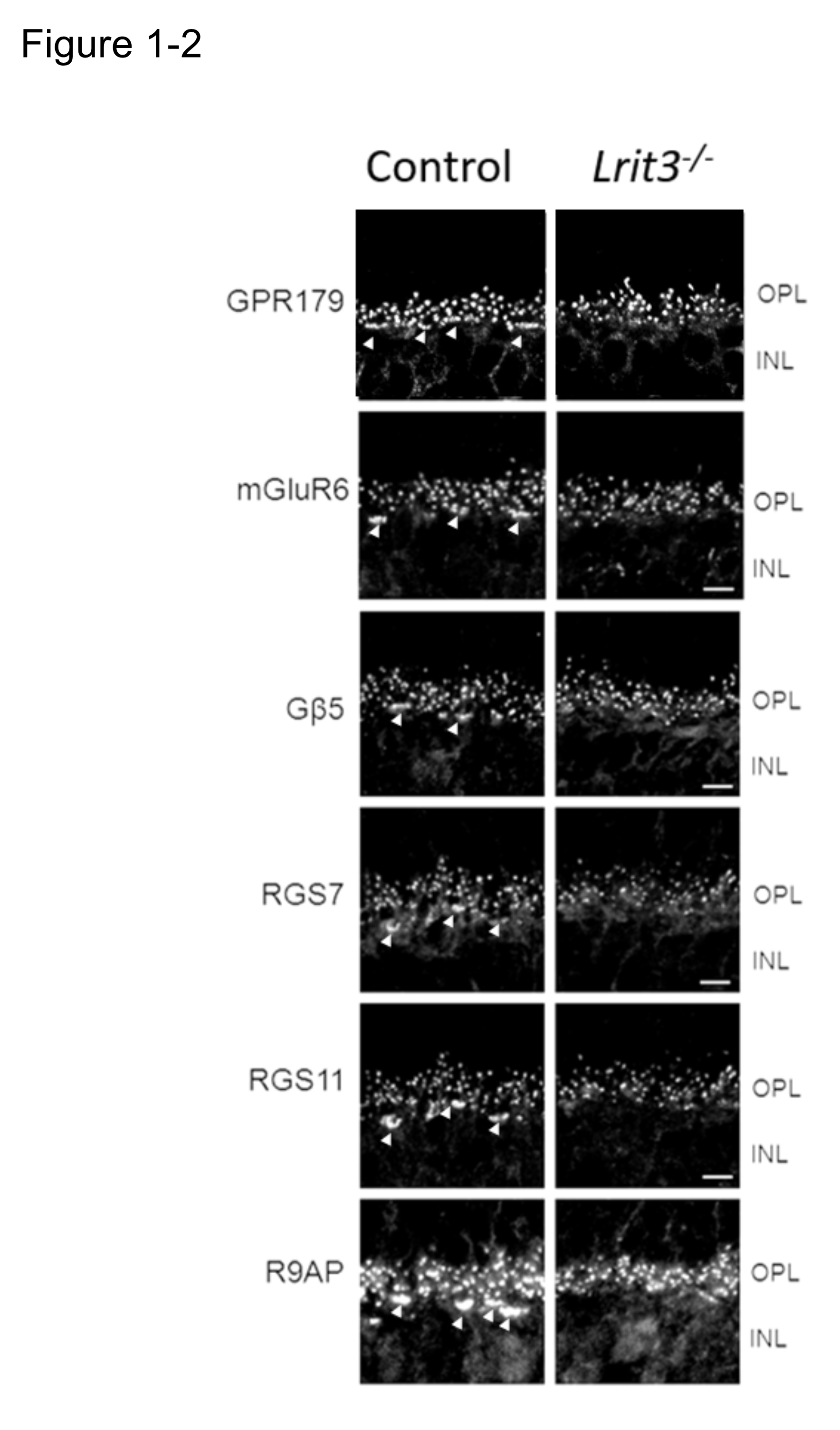

Supplement: Extended Data Figure 1-2 — The absence of LRIT3 has differential effects on rod and cone DBC signalplex proteins. Immunohistochemical staining for GPR179, mGluR6, Gβ5, RGS7, RGS11, and R9AP show punctate staining at the dendritic tips of both rod and cone (large clusters at the base of the OPL and indicated by arrowheads) DBCs in control mice. In Lrit3-/- mice these proteins are localized on the rod DBC dendritic tips but are absent from the cone DBCs. Note the lack of the large clusters at the bottom of the OPL. Scale bar = 5 μm. INL, inner nuclear layer. Scale bar = 5 μm. Download Figure 1-2, TIF file. [file sup_enu-eN-NWR-0002-20-s02.tif]

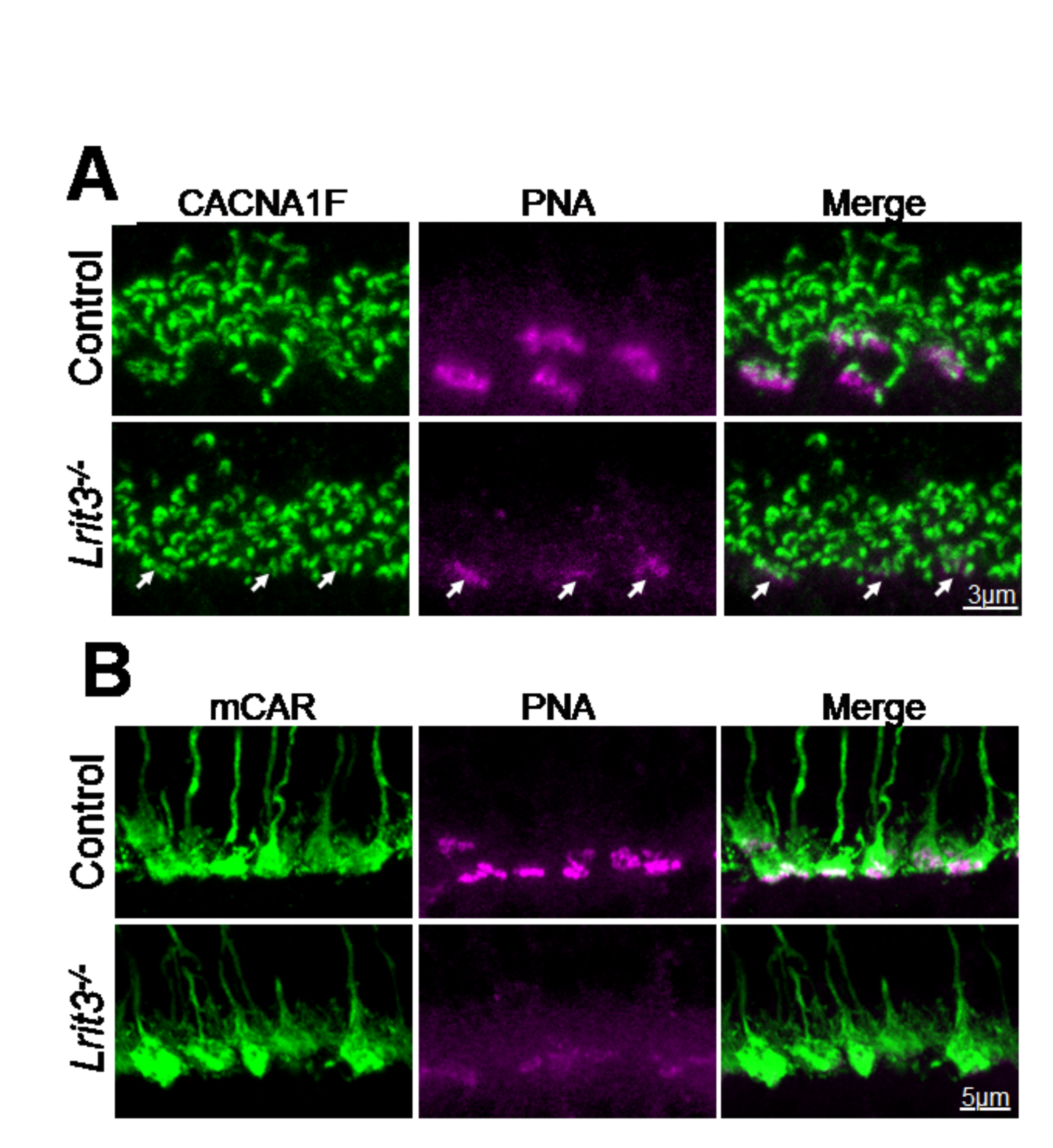

Supplement: Extended Data Figure 2-1 — Cone terminal appear normal in Lrit3-/-retinas. A, Immunohistochemical staining for the presynaptic markers CACNA1F and PNA. CACNA1F staining in Lrit3-/- retinas is indistinguishable from controls. PNA staining in Lrit3-/- retinas is decreased, but not completely absent. B, Staining for the cone terminal marker mCAR (cone arrestin) and PNA. mCAR staining in the Lrit3-/- retinas is similar to controls, and PNA is decreased in Lrit3-/- retinas. Download Figure 2-1, TIF file. [file sup_enu-eN-NWR-0002-20-s03.tif]
